# Supplementary material for: Causal Relationship between Aging and Anorexia Nervosa: A White-Matter-Microstructure-Mediated Mendelian Randomization Analysis
Source: Biomedicines. 2024 Aug 16;12(8):1874. doi: 10.3390/biomedicines12081874 (PMC11351342; doi:10.3390/biomedicines12081874)
Supplement: Supplementary file 1 [file biomedicines-12-01874-s001.zip › Supplementary Figure/Figure S9/Leaveoneout plot of MR analysis for ubm-b-1511 to AN.pdf]

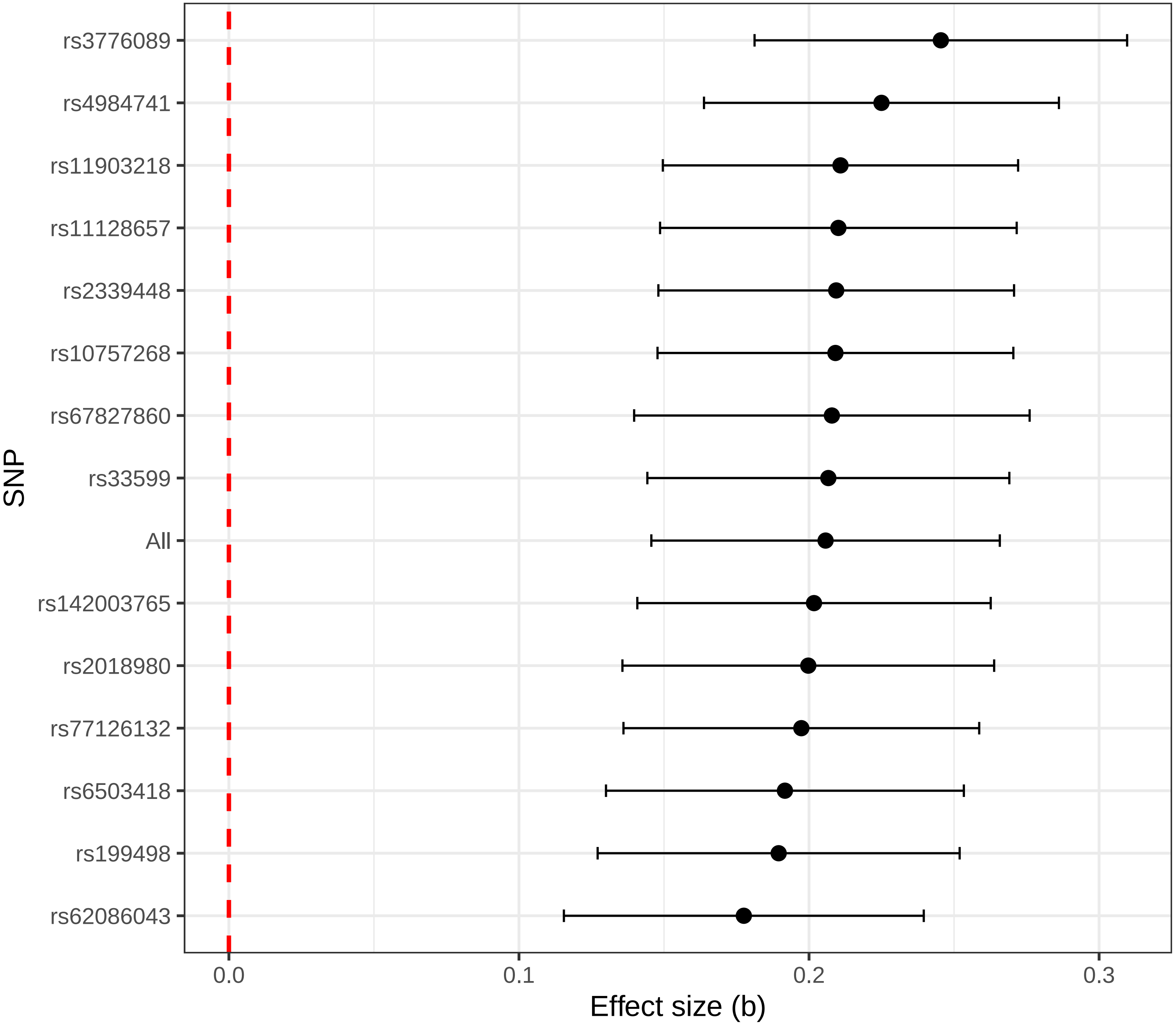

rs3776089
rs4984741
rs11903218
rs11128657
rs2339448
rs10757268
rs67827860
rs33599
All
rs142003765
rs2018980
rs77126132
rs6503418
rs199498
rs62086043
SNP
0.0
0.1
0.2
0.3
Effect size (b)
